# Second Human Pegivirus in Hepatitis C Virus–Infected and Hepatitis C Virus/HIV-1–Co-infected Persons Who Inject Drugs, China

**DOI:** 10.3201/eid2405.161162

**Published:** 2018-05

**Authors:** Haiying Wang, Zhengwei Wan, Qiang Sun, Nalin Zhu, Tianyi Li, Xuqi Ren, Xiaoping An, Shuyun Deng, Yue Wu, Xiufen Li, Lin Li, Jingyun Li, Yigang Tong, Shixing Tang

**Affiliations:** Southern Medical University, Guangzhou, China (H. Wang, Z. Wan, N. Zhu, Y. Wu, X. Li, S. Tang);; Guangdong Provincial Key Laboratory of Tropical Disease Research, Guangzhou (H. Wang, Z. Wan, N. Zhu, Y. Wu, X. Li, S. Tang);; Beijing Institute of Microbiology and Epidemiology, Beijing, China (Q. Sun, T. Li, X. An, L. Li, J. Li, Y. Tong);; Guangdong Provincial Dermatology Hospital, Guangzhou (X. Ren);; Nanfang Hospital, Guangzhou (S. Deng)

**Keywords:** second human pegivirus, HPgV-2, HHpgV-1, hepatitis C virus, HCV, HIV-1, HPgV, viruses, men who have sex with men, MSM, persons who inject drugs, PWID, China, pegivirus

## Abstract

We report the presence of the second human pegivirus (HPgV-2) in Guangdong and Sichuan Provinces in China. The prevalence of HPgV-2 in hepatitis C virus/HIV-1–co-infected persons who inject drugs was 12.9% in Guangdong and 15.9% in Sichuan. This population is at high risk for HPgV-2 infection.

In 2015, the second human pegivirus (HPgV-2) was independently reported by 2 groups in the United States (*1*,*2*). Previous reports have indicated that HPgV-2 (also known as HHpgV-1) is a transfusion-transmitted virus and is associated with hepatitis C virus (HCV) infection (*1*–*5*). The distribution and prevalence of HPgV-2 infection worldwide are of great importance but remain to be determined. In this study, we demonstrate the existence of HPgV-2 in the southern province of Guangdong and southwestern province of Sichuan in China. We have also identified HCV-infected persons, in particular HCV/HIV-1 co-infected persons who inject drugs (PWID), as populations at high risk for HPgV-2 infection. In addition, our work reveals the difference in the prevalence, distribution, and phylogeny between the first human pegivirus (HPgV; formerly GB virus C or hepatitis G virus) (*6*,*7*) and HPgV-2.

## The Study

**Figure 1:**
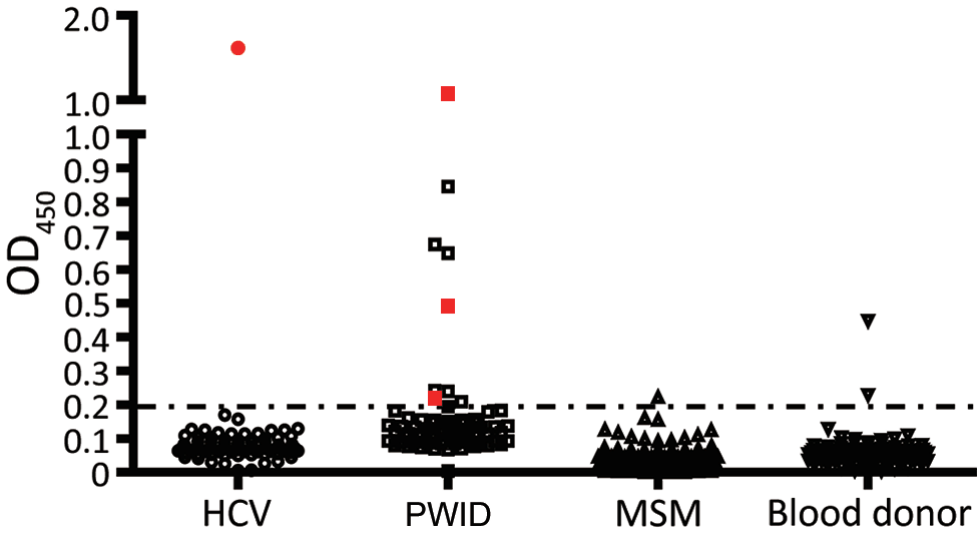
Detection of second human pegivirus (HPgV-2) antibodies in different samples in Guangdong and Sichuan Provinces, China. Serum or plasma samples from 86 HCV-infected patients, 70 PWID, 122 MSM, and 102 blood donors (100 samples that were negative for HPgV-2 antibodies plus 2 positive samples) are included. The antibody titers from each sample are plotted on the *y*-axis. HPgV-2 RNA–positive samples are shown in red. HCV, hepatitis C virus; MSM, men who have sex with men; OD_450_, optical density at 450 nm; PWID, persons who inject drugs.

In our initial investigation of HPgV-2, we screened a total of 367 delinked serum or plasma samples from high-risk groups for infection with HCV and HIV-1 and 500 healthy volunteer blood donors from Guangdong Province, China, by using ELISA (*2*,*5*), and a nested reverse transcription PCR targeting both the 5′ untranslated region and nonstructural protein 3 regions of HPgV-2 (*3*,*5*). We observed a low frequency (0.4%) of HPgV-2 antibody detection and the absence of HPgV-2 viremia in healthy blood donors tested in our study. Out of 86 HCV-infected patients, 1 (1.2%) was positive for both HPgV-2 antibodies and viral RNA (Table 1). Furthermore, we did not detect HPgV-2 RNA in men who have sex with men (MSM), although 1 (0.5%) of the 211 MSM was weakly positive for HPgV-2 antibodies and negative for HPgV-2 RNA (Table 1; Figure 1).

**Table 1:** Detection frequencies of HPgV-2 in different populations in Guangdong and Sichuan Provinces, China*

Province, group, and subgroup	No. tested	HPgV-2		HPgV RNA+, no. (%)
		Ab+, no. (%)	RNA+, no. (%)	
Guangdong Province				
HCV-infected patients				
Ab+/RNA+	57	1 (1.8)	1(1.8)	8 (14.0)
Ab–/RNA+	7	0	0	0
Ab+/RNA–	22	0	0	4 (18.2)
Total	86	1 (1.2)	1 (1.2)	12 (14.0)
PWID				
HIV-1+/HCV Ab+/RNA+	70	9 (12.9)	4 (5.7)	28 (40.0)
MSM				
HIV-1+/HCV+	12	1 (8.3)	0	4 (33.3)
HIV-1+/HCV–	100	0	0	28 (28.0)
HIV-1–/HCV+	10	0	0	1 (10.0)
HIV-1–/HCV–	89	0	0	7 (7.9)
Total	211	1 (0.5)	0	40 (19.0)
Blood donors				
HIV-1–/HCV–/HBV–	500	2 (0.4)	0	NT
HCV Ab+/HCV RNA+	2	2	2	NT

Sichuan Province				
PWID				
HIV-1+/HCV Ab+	270	43 (15.9)	8 (3.0)	NT

*Ab, antibodies; HBV, hepatitis B virus; HCV, hepatitis C virus; HPgV, human pegivirus; HPgV-2, second human pegivirus; MSM, men who have sex with men; NT, not tested; PWID, people who inject drugs; +, positive; –, negative.

We observed a relatively high prevalence of HPgV-2 infection in HCV/HIV-1 co-infected PWID in Guangdong Province; 12.9% (9/70) were positive for HPgV-2 antibodies and 5.7% (4/70) for HPgV-2 RNA (Table 1). We obtained similar results from 270 PWID from Sichuan Province; 15.9% (43/270) were positive for HPgV-2 antibodies and 3.0% (8/270) for HPgV-2 RNA (Table 1). Using the Fisher exact test, we observed a statistically significant difference between HCV-positive and HCV-negative patients in the prevalence of having HPgV-2 antibodies (6.2% vs. 0; p<0.001) and prevalence of having HPgV-2 RNA (5% vs. 0; p = 0.026). Similarly, we observed a statistically significant difference between HIV-1–positive/HCV-positive patients and HIV-1–positive/HCV-negative patients in the prevalence of having HPgV-2 antibodies (10% vs. 0; p<0.001) and prevalence of having HPgV-2 RNA (4% vs. 0; p = 0.040) (Table 2). These findings indicate a close association between HPgV-2 and HCV infection and synergy between HIV-1 and HCV infection with respect to HPgV-2 infections (*5*).

**Table 2:** Comparison of HPgV and HPgV-2 infections among HCV-, HIV-1–, and HIV-1/HCV–infected populations in Guangdong Province, China*

Group	No. tested	HPgV-2 Ab+			HPgV-2 RNA+			HPgV RNA+	
		No. (%)	p value		No. (%)	p value		No. (%)	p value
HCV									
+	178	11 (6.2)	<0.001		5 (2.8)	0.026		45 (25.3)	0.130
–	189	0 (0)			0 (0)			35 (18.5)	
HIV-1									
+	182	10 (5.5)	0.005		4 (2.2)	0.212		60 (33.0)	<0.001
–	185	1 (0.5)			1 (0.5)			20 (10.8)	
HIV-1/HCV									
+/+	82	10 (12.2)	<0.001		4 (4.9)	0.040		32 (39.0)	0.154
+/−	100	0 (0)			0 (0)			28 (28.0)	

*p values calculated by using Fisher exact test. Ab, antibodies; HCV, hepatitis C virus; HPgV, human pegivirus; HPgV-2, second human pegivirus; +, positive; –, negative.

**Figure 2:**
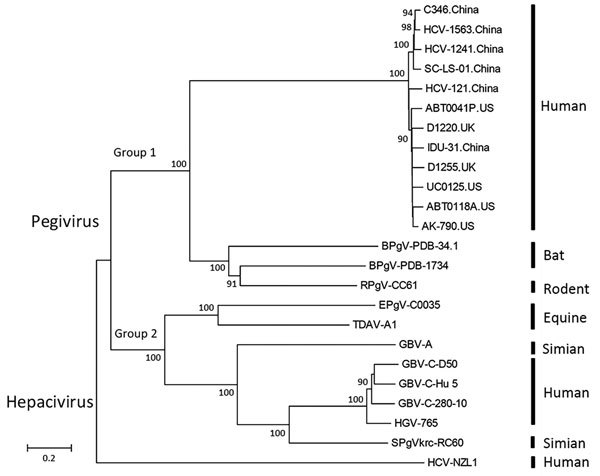
Phylogenetic analysis of second human pegivirus (HPgV-2) isolates identified in our study (China) and abroad (UK and US). Phylogenetic trees of nucleotide sequences from complete sequences of HPgV-2 strains isolated in our study and elsewhere as well as hepatitis C virus and pegivirus strains from humans, simians, equids, bats, and rodents are included. The phylogenetic trees were constructed with the neighbor-joining tree method using MEGA6 software (http://www.megasoftware.net). Bootstrap analysis with 1,000 replicates was performed to determine the robustness of branching; values are shown on branches. Scale bar indicates the estimated number of nucleotide substitutions per site. The near full-length genome sequences of HPgV-2 identified in this study have been submitted to GenBank under accession numbers KX528230 (HCV-121), KX528231 (IDU31), KY971606 (C346), MG457178 (SC-LS-01), MF770985 (HCV1241), and MF770986 (HCV1563). UK, United Kingdom; US, United States.

Furthermore, we obtained 6 near full-length genome sequences of HPgV-2 by using next-generation sequencing or sequencing of PCR products (*5*). These strains from China, which included 2 from PWID (IDU31 and SC-LS-01), 2 from HCV-infected patients (HCV-121 and C346), and 2 from HCV-infected blood donors (HCV1241 and HCV1563), exhibited an identity of 93.6%–97.8% at the whole-genome level. Compared with other HPgV-2 strains from the United States and United Kingdom, the nucleotide sequence identity was 93.7%–96.2%. Sequence divergence was greatest at synonymous sites, with ratios of nonsynonymous to synonymous nucleotide substitutions of 0.125–0.150, which are consistent with other reports (*1*–*3*). Phylogenetic analysis indicated that HPgV-2 strains from China, the United States, and the United Kingdom clustered together to form a separate branch and fell into group 1 with the closely related pegiviruses from bats and rodents (Figure 2). Other pegiviruses from human, simian, and equine sources formed group 2, in which the variants of HPgV fell into a separate clade. These results illustrate the difference between the 2 human pegiviruses (*1*,*2*,*8*) and the low level of genetic diversity of HPgV-2 strains (*1*–*3*).

In contrast to our findings on HPgV-2 infection, we observed a high frequency of HPgV infection across all 3 populations tested (HCV-infected patients, PWID, and MSM) (Tables 1, 2). The percentage of HPgV viremia was 14.0% (14/86) in HCV-infected patients, 19.0% (40/211) in MSM, and 40.0% (28/70) in PWID (Table 1). Among MSM, the prevalence of HPgV RNA was 28.0% (28/100) in those who were infected with HIV-1 alone and 33.3% (4/12) in those who were HIV-1/HCV co-infected (Table 1). For MSM who were negative for both HIV-1 and HCV, 7.9% (7/89) were positive for HPgV RNA (Table 1).

## Conclusions

We report the detection of the second human pegivirus, HPgV-2, in HCV-infected (in particular HCV/HIV-1 co-infected) persons in Guangdong and Sichuan Provinces, China (Table 1). Our results and those from previous studies demonstrate that the virus occurs in several geographically distinct regions in the world (*1*–*4*,*9*,*10*).

HPgV and HPgV-2 are the only known human pegiviruses (*8*), and comparing their association with HCV and HIV-1 infection is of great interest. Consistent with previous reports, we found that the prevalence of HPgV viremia was 7.9% in HCV and HIV-1–negative MSM and 33.3%–40% in HCV/HIV-1 co-infected MSM and PWID (Table 1). In contrast, only 0.5% of MSM and 0.4% of healthy blood donors were positive for HPgV-2 antibodies, but all were negative for HPgV-2 RNA (Table 1). These results indicate that HPgV-2 infection might be much less frequent than HPgV infection, possibly because of its low transmissibility or high clearance rate (*2*–*4*). The dramatic difference of distribution and prevalence between HPgV and HPgV-2 infections in different populations provides a clue for investigation of disease association with HPgV-2. HPgV does not cause human diseases (*11*) and can inhibit HIV-1 replication as well as prolong survival of HIV-1–infected and Ebola virus–infected patients (*12*–*14*). However, possible pathogenicity and disease association of HPgV-2 remain to be elucidated.

The high-risk populations susceptible to HPgV-2 infection include HCV-infected patients and, in particular, HCV/HIV-1 co-infected PWID. Most (93.3%) of HPgV-2 infected patients were also co-infected with HCV (*1*–*4*). Notably, the relatively high frequency of HPgV-2 RNA detection was observed in HCV/HIV-1 co-infected PWID in Guangdong (5.7%) and Sichuan (3.0%) Provinces of China (Table 1) and in the United States (10.9%) (*9*). In contrast, a somewhat lower percentage (1.7%) of HCV-positive PWID in the United Kingdom were reported to be HPgV-2 RNA positive, whereas none of the 30 HIV-1 singly infected and 36 HCV/HIV-1 co-infected PWID were positive for HPgV-2 RNA (*3*). These discordant results warrant more studies in different countries to address the association between HPgV-2 and HCV/HIV-1 co-infection.

Our findings are subject to 2 limitations. First, because a limited number of samples from only 2 provinces of China were tested, the results might not represent overall prevalence of HPgV-2 infection throughout all of China. Second, this study was a cross-sectional rather than a longitudinal study, therefore, the proportions of persistent infection and natural history of HPgV-2 infection remain to be determined.

Future studies should address several questions: whether the close association between HPgV-2 and HCV infection represents a biologic dependence of these 2 viruses; how HCV/HIV-1 co-infection facilitates HPgV-2 infection; and whether HCV or HIV-1 viral proteins enhance the transmissibility or infectivity of HPgV-2. In addition, because the rarity of HPgV-2 detection in MSM could be a result of the low frequency of HCV or HIV-1 infection or the transmission route of HPgV-2, further research should aim to determine if HPgV-2 is more like a transfusion-transmitted virus rather than a sexually transmitted virus.
